# Massive fetal goiter treated by intra-amniotic injection of levothyroxine: a case report

**DOI:** 10.1515/crpm-2024-0006

**Published:** 2024-05-27

**Authors:** Nami Tamura, Yuka Yamamoto, Jun Takeda, Junpei Ishigami, Hiroki Suganuma, Toshiaki Shimizu, Atsuo Itakura

**Affiliations:** Department of Obstetrics and Gynecology, 2012847 Juntendo University Faculty of Medicine, Tokyo, Japan; Department of Pediatrics, 2012847 Juntendo University Faculty of Medicine, Tokyo, Japan

**Keywords:** cordocentesis, fetal therapy, fetal goitrous hypothyroidism

## Abstract

**Objectives:**

Fetal dyshormonogenetic goiter is a rare condition associated with perinatal complications and sequelae in infants. Although prenatal management remains controversial, further evidence is required for optimal management.

**Case presentation:**

A 30-year-old pregnant woman with no history of thyroid disease was referred to our hospital with polyhydramnios. Fetal ultrasonography revealed fetal goiter. Cordocentesis revealed increased thyroid-stimulating hormone (TSH) and low levels of free thyroxine 4 (fT4), which was the basis of diagnosis of fetal hypothyroidism. Intra-amniotic injections of levothyroxine were administered, resulting in a reduction in the goiter size, amount of amniotic fluid, and level of maternal TSH. The mother was euthyroid during pregnancy. The infant was delivered vaginally at full term with a normal thyroid size and no respiratory disorders except hypothyroidism. At 2 years of age, her neurodevelopment is normal.

**Conclusions:**

Intra-amniotic injections of levothyroxine for fetal hypothyroidism with massive goiter and polyhydramnios may improve perinatal outcomes.

## Introduction

The incidence of fetal goiter is reported to be 1 in 40,000 deliveries [1]. The causes of fetal goiter vary, including hyperthyroidism, hypothyroidism, and euthyroid states [2]. Thus, assessment of fetal thyroid function is important when considering *in utero* treatment, and cordocentesis is the most accurate method to evaluate fetal thyroid hormone levels for thyroid function [3].

Enlarged fetal goiters are associated with a risk of preterm birth because of polyhydramnios, fetal malrotation, and respiratory disorders immediately after birth because of the tracheal and esophageal compression. Therefore, prenatal diagnosis and treatment should be considered; however, a treatment plan has not yet been established [4]. In previous reports, the intra-amniotic injection of levothyroxine is significantly effective in reducing the size of fetal goiter without major complications [5]. The increased risk for preterm birth was reported up to 9.7 %. In choosing the treatment of intra-amniotic injection, the basic risk of amniocentesis such as infection, fetal bleeding, fetal bradycardia, premature labor, and fetal death must be considered [6]. Only one case reported the intrauterine death after triiodothyronine (T3) and thyroxine (T4) injection to the euthyroid mother [7]. Although the fetal treatment is significantly effective, there has been no evidence for the long-term outcome.

Herein, we present a case of fetal goitrous hypothyroidism treated *in utero* with successful vaginal delivery at term without any respiratory complications and good long-term outcome.

## Case presentation

**Figure 1:**
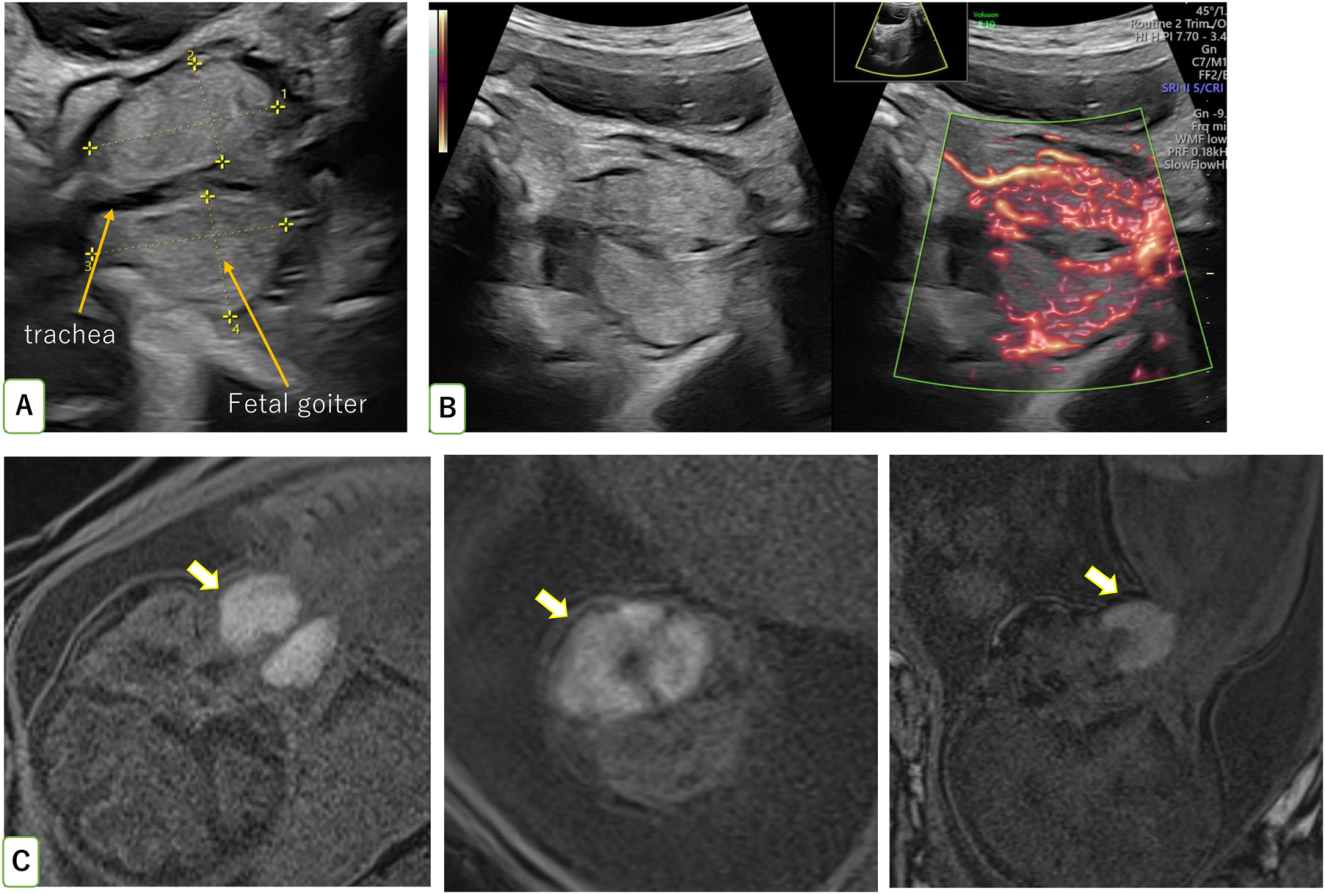
Ultrasonographic examination and magnetic resonance imaging (MRI) of the fetal goiter at 33 weeks’ gestation. (A) Size of the right thyroid lobe was 2.9 × 1.6 cm and that of the left thyroid lobe was 2.9 × 1.9 cm in the anterior aspect of the fetal neck. (B) On color Doppler examination, the vascularization of the goiter was concentrated in the periphery. (C) On fetal magnetic resonance imaging (MRI) T1-weighted image shows a massive fetal goiter.

A 30-year-old woman with her first pregnancy was referred to our hospital with polyhydramnios at 31 weeks’ gestation. She had no past or family history of thyroid disease, and her iodine intake was normal. The thyroid hormone levels of the mother at her first visit to our hospital were normal (low level of thyroid-stimulating hormone (TSH): 0.7 mIU/L, free thyroxine 4 (fT4): 1.2 ng/dL, TRAb negative). Her pregnancy was conceived by artificial insemination by her husband (AIH). She underwent hysterosalpingography (HSG) using an oil-soluble iodinated contrast medium and AIH was performed immediately after her menstrual period. She underwent a pregnancy check-up at a nearby clinic, and polyhydramnios was diagnosed at 32 weeks’ gestation. The patient was referred to our hospital because of polyhydramnios. A detailed ultrasound examination revealed a massive fetal goiter with the right and left lobe measuring 2.9 × 1.6 cm and 2.9 × 1.9 cm, respectively, in the anterior aspect of the fetal neck, which was compressing the trachea and esophagus (Figure 1A). Color Doppler analysis showed that the vascularization of the goiter was concentrated in the peripheral region (Figure 1B). The amniotic fluid had an amniotic fluid index (AFI) of 34. No other fetal anomalies, such as delayed bone maturation or fetal tachycardia, were detected. The fetal goiter was also visualized as high intensity on the fetal magnetic resonance imaging (MRI) T1 signal (Figure 1C). The iodine level of the mother’s urine was 125,040 µg/24 h which was extremely high compared to the normal level in Japanese women (median value 346 µg/24 h) [8]. To accurately evaluate fetal thyroid function and clarify the cause of the fetal goiter, cordocentesis was performed at 33 weeks’ gestation. TSH level in the umbilical cord blood was 212 mIU/L (normal 0.35–4.94 mIU/L) which was highly elevated, whereas the fT4 level was decreased to 0.5 ng/dL (normal 0.7–1.48 ng/dL). The TSH level was also elevated at 2.8 mIU/L (normal 0.15–0.55 mIU/L) [9] and the fT4 level was 0.3 ng/dL in the amniotic fluid. Based on these findings, the cause of the fetal goiter was diagnosed as fetal goitrous hypothyroidism.

After approval from the ethics committee, fetal therapy with intra-amniotic injection of 200 µg levothyroxine (T4) was performed every week from 34 weeks’ gestation until the size of the fetal goiter decreased to the 95th percentile to avoid complications at birth. TSH, fT3, and fT4 levels in the amniotic fluid were evaluated at the time of fetal therapy. The size of the goiter reduced from 8.49 to 3.5 cm^2^ (the image of transverse thyroid was obtained at the trachea placed exactly on the midline, and the measurement of the fetal thyroid area (FTA) was performed by drawing the outline manually [10]) and AFI decreased from 34 to 9.2. After four injections of levothyroxine, the fetus was suspected to be ready for birth, and treatment was supposed to be administered after birth, if needed.

Labor was induced by oxytocin at 38 weeks and 4 days of gestation and a 2,742 g female infant was delivered vaginally without any complications, including respiratory disorders. The Apgar scores were eight and nine at 1 and 5 min, respectively, and the umbilical arterial blood gas pH was 7.277. The thyroid hormone levels of the mother after 4 days from delivery were still normal (TSH: 0.87 mIU/L, fT4: 1.2 ng/dL). Thyroid hormone levels from umbilical cord blood at birth were TSH 49.2 mIU/L, fT4 1.1 ng/dL. Thyroid hormone levels of the baby’s blood were TSH 133.0 mIU/L, fT4 1.2 ng/dL, and the iodine level of the baby’s urine was 1,425 µg/mgCre which was extremely high compared to the normal level in Japanese neonates (median value 121.0 µg/mgCre). The size of the thyroid on ultrasonography was within the normal range, measuring 2.7 × 1.1 cm, and both knee epiphyses were present on radiography. TSH level of the baby was decreasing steadily, but the decrease slowed down at day 21 (TSH 34.0 mIU/L, fT4 1.0 ng/dL), showing subclinical hypothyroidism, and levothyroxine medication was started at the dose of 10 μg/kg and increased to 25 μg/kg at 3 months of age. The medication was continued until 10 months of age. At 2 years of age, her thyroid level is normal without any enlarged thyroid with no medications. Her growth and neurological development are within the normal range.

## Discussion and conclusions

Optimal management of fetal goiter has not yet been established, although several successful cases of fetal therapy have been reported. There are two issues in the discussion of fetal goiter: diagnosis, fetal therapy and outcomes.

Regarding the diagnosis, Huel’s ultrasonographic score is one of the options to evaluate fetal goiter [11]. Huel et al. gathered the ultrasound features of fetal hyperthyroidism and hypothyroidism, including bone maturation, fetal heart rate, fetal movements, and vascularization with color Doppler. In the present case, color Doppler ultrasound showed vascularization of the goiter concentrated in the peripheral region. Fetal heart rate, fetal bone maturation (assessed by the presence of bilateral knee epiphysis on knee radiography), and fetal movement were all normal, accounting for one point on Huel’s ultrasound score, suggesting fetal hypothyroidism. However, a more accurate assessment of thyroid function is required when considering fetal treatment. Cordocentesis can directly assess fetal thyroid function but is more invasive than amniocentesis and carries risks such as bleeding, cord hematoma, and most importantly, fetal loss in about 1–4 % of cases [12]. According to previous studies, there are some discrepancies between the levels in the amniotic fluid and cord blood of fT4 [13], but not TSH [14]. Indeed, the cordocentesis in the present case showed an obvious decrease in fT4 levels and an increase in TSH levels, which resulted in the diagnosis of fetal hypothyroidism.

The treatment of fetal goiter has not been established; however, several previous reports have confirmed the feasibility and safety of intra-amniotic treatment [4]. *In utero*, an enlarged goiter may cause polyhydramnios, fetal malrotation, and respiratory disorders at birth secondary to tracheal compression [15]. Long-term issues associated with congenital hypothyroidism includes neurological and intellectual dysfunction and slow growth [5]. Persistent TSH elevation is a risk factor for developmental disorders in infants [16]. It is recommended that medication be initiated immediately, at least within 14 days of birth, for neonatal hypothyroidism. Regarding short-term outcomes, we achieved a size reduction of the fetal goiter, which led to a normal amniotic fluid level, decreased compression of the trachea and esophagus, and vaginal delivery without complications. Regarding long-term prognosis, some reports have shown no adverse or normal neurodevelopmental outcomes [17]. Collection of favorable outcomes with fetal therapy is important to establish a standard. In the present case, the baby needed the medication from day 20 to 10 months of age, but the neurological development has been favorable.

It is important to keep in mind that when polyhydramnios is detected, an enlarged goiter may be the cause; if so, treatment may be possible. Perinatal complications may be prevented by fetal treatment. The present case showed a favorable outcome for both short- and long-term prognosis at 2 years of age with fetal treatment. More evidence is needed to determine the long-term prognosis of fetal treatment of fetal goiters.
